# Programmable gravitational valves in idiopathic normal pressure hydrocephalus: long-term outcomes after a 3-year follow-up

**DOI:** 10.1007/s00701-025-06563-y

**Published:** 2025-05-24

**Authors:** Stefano Colonna, Carla Paracampo, Elena Garro, Enrico Lo Bue, Alberto Morello, Alessandro Pesaresi, Luca Ceroni, Salvatore Petrone, Diego Garbossa, Fabio Cofano, Alessandro Fiumefreddo

**Affiliations:** 1 Neurosurgery Unit, Department of Neuroscience “Rita Levi-Montalcini”, University of Turin, Via Cherasco, 15, 10126 Turin, Italy; 2 Department of Psychology, University of Turin, Turin, Italy

**Keywords:** Idiopathic normal pressure hydrocephalus, Gravitational valve, Adjustable pressure valve, Ventriculoperitoneal shunt

## Abstract

**Background:**

The development of shunting valve technologies for the surgical treatment of idiopathic normal pressure hydrocephalus (iNPH) has advanced significantly over the decades, with adjustable gravitational valves (GV) emerging as a promising alternative to traditional fixed-pressure valves. This study aimed to investigate the safety and effectiveness of adjustable GV for the surgical treatment of iNPH after a 3-year follow-up.

**Methods:**

Adult patients treated with ventriculoperitoneal shunt (VPS) using programmable GVs were retrospectively evaluated. Neurological outcome was assessed according to the iNPH Grading Scale (INPHGS). Postoperative early and late complications, pre- and post-implantation pressure settings, and type and number of post-implantation pressure adjustments were recorded at each follow-up.

**Results:**

A total of 76 patients were evaluated, with a median postoperative follow-up of 36 (24–42) months. The mean preoperative and postoperative iNPHGS scores were 4.2 ± 1.6 and 3.5 ± 1.5, respectively, demonstrating a significant overall clinical improvement after VPS surgery (*p* < 0.001). Overall, 7 (9.2%) patients required surgical intervention due to late complications. No cases of valve dysfunction were reported. During follow-up, 54 (71.1%) patients underwent valve setting adjustments, with a median number of post-implantation valve setting modifications of 1. No significant associations were found between postoperative outcomes and preoperative characteristics including age and initial opening pressure parameters.

**Conclusions:**

Adjustable GVs are a safe and effective alternative to traditional fixed differential pressure valves for the surgical treatment of iNPH. After a 3-year follow-up, the overall postoperative complication rate was acceptable, with a significantly lower rate of valve dysfunction compared to previous literature findings.

## Introduction

Idiopathic normal pressure hydrocephalus (iNPH) is a potentially reversible neurological condition characterized by the triad of gait disturbance, cognitive impairment, and urinary incontinence, often presenting in elderly populations [8]. The primary treatment involves cerebrospinal fluid (CSF) shunting, with ventriculoperitoneal shunt (VPS) being the most used procedure [5, 9, 14]. The development of shunting valve technologies has advanced significantly over the decades, driven by the need to optimize CSF drainage and minimize complications. Early fixed differential pressure (FDP) valves, while effective in relieving symptoms, often led to overdrainage-related complications such as subdural hematomas and slit ventricle syndrome. To address this issue, adjustable flow-regulated valves (FV) with a differential pressure unit (DPU) component were introduced, allowing for noninvasive post-implantation adjustments to better match individual patient-specific needs [3, 18].

More recently, gravitational units (GU) have been integrated into FV valve designs to address position-dependent drainage variability and to reduce overdrainage complications. These advancements have enhanced the safety and adaptability of shunting systems, though challenges remain in balancing cost, complexity, and long-term outcomes [2]. Moreover, several studies have demonstrated lower failure rates and reduced shunt revision surgeries with gravitational valves (GV) compared to FDP valves, although the advantages of one over the other remain debated [17, 27].

Despite their theoretical superiority, the use of GV raises practical and economic questions. Adjustable GV provide flexibility in optimizing CSF drainage postoperatively, but their increased complexity and higher cost compared to FDP valves may limit accessibility and cost-effectiveness. To date, evidence from the literature regarding safety, effectiveness, and long-term durability of antigravitational valves remains limited [4, 7, 10, 21, 22, 25]. The aim of this study is to evaluate the clinical outcomes associated with adjustable GV in the surgical treatment of iNPH based on a single-center experience with a long-term follow-up. This study seeks to provide evidence to guide clinical decision-making and optimize treatment strategies for patients with iNPH.

## Methods

In this single-center observational study, adult patients diagnosed with probable iNPH and treated with VPS using adjustable gravitational valves from January 2018 to December 2023 were retrospectively evaluated. Patients with coexisting or alternative etiologies of hydrocephalus other than iNPH, those previously treated with CSF diversion or shunt surgery, or those treated with other types of valves were excluded from the study.

This study was conducted in accordance with the Guidelines for Good Clinical Practice and the Declaration of Helsinki of the World Medical Association. Written informed consent was regularly obtained from patients for diagnostic and surgical procedures.

### Preoperative evaluation

All patients included in the study were preoperatively diagnosed with “possible” iNPH based on the criteria defined by the Guidelines for the Management of Idiopathic Normal-Pressure Hydrocephalus recommended by the Japanese Society of Normal Pressure Hydrocephalus [19].

A preoperative lumbar tap test (TT) was performed in all patients, with the evaluation of time-up and go test (TUGT) and 10-m walking test (10MWT) performances before and one hour after the TT. Following hospital discharge, all patients maintained a daily diary documenting any subjective improvement in neurological symptoms during the week after the TT. Eligibility for VPS surgery was considered for patients who demonstrated significant clinical improvement in TUGT and 10MWT performance after the TT, with subjective clinical improvements from the daily reports considered as supportive evidence favoring VPS treatment.

Adjustable differential pressure valves with an adjustable gravitational unit (M.blue plus®, Miethke, Potsdam, Germany) were implanted in all patients. The initial valve pressure settings for both DPU and GU were determined by a senior neurosurgeon based on each patient’s clinical and radiological features. Specifically, factors such as patient height, activity level, and conditions that could increase intra-abdominal pressure, such as obesity, were considered when calculating the GU opening pressure. When necessary, a noninvasive method was available for post-implantation pressure adjustments, allowing modifications to both the DPU and GU valve settings in accordance with the desired clinical target.

### Postoperative evaluation

A head CT scan, skull X-ray, and two-projection abdominal X-ray were performed on the first postoperative day to evaluate proximal and distal catheter positioning and valve pressure settings. Postoperative follow-up included a clinical neurological examination, and a head CT scan performed two months after VPS surgery. The first follow-up examination was scheduled six months post-surgery and subsequently continued every 6 to 12 months based on clinical and radiological findings.

All patients were clinically evaluated preoperatively and postoperatively using the iNPH Grading Scale (iNPHGS) [11]. Hakim’s triad symptoms were assessed both before surgery and at each follow-up evaluation. Overall neurological outcome improvement was defined as a reduction of ≥ 1 point in the postoperative iNPHGS score compared to the preoperative assessment.

The settings of both DPU and GU of the implanted valves were evaluated at each follow-up and compared to the initial valve configuration. If applicable, the number and type of post-implantation valve setting modification were recorded for each patient. The decision to modify the initial valve settings – either for one or both valve components – was assessed at each follow-up examination based on clinical and radiological data. Postoperative complication, including proximal or distal catheter obstruction, shunt catheter displacement, shunt infection, and subdural hematoma/hygroma were reported. Early complications were defined as those occurring during the postoperative hospitalization, while late complications were those occurring during follow-up.

### Statistical analysis

Descriptive statistics were reported as mean and standard deviation for continuous variables or as frequency and percentage for qualitative variables. The Chi-squared test was used to evaluate the association between two qualitative variables. When necessary (> 20% of values ≤ 5 and/or the presence of values < 1), Cramer’s Phi and V coefficients were used to verify the association between variables. The Kolmogorov–Smirnov test was used to determine the statistical normality of a quantitative variable. Spearman’s Rho correlation test was used to assess the correlation between two non-parametric quantitative variables. Mann–Whitney U test was used to evaluate differences in a non-parametric quantitative variable in relation to a two-category qualitative variable. Statistical significance was defined as a p-value ≤ 0.05. All statistical analyses were performed using SPSS Statistics software (IBM SPSS Statistics for Windows, Version 28.0; IBM Corp., Armonk, New York, USA).

## Results

### Study population

A total of 76 patients – 23 (30.3%) females and 53 (69.7%) males – met the inclusion criteria and were evaluated. The mean age at the time of VPS surgery was 72.9 ± 9.9 years, with a median postoperative follow-up of 36 (24–42) months. Mean preoperative and postoperative iNPHGS scores were 4.2 ± 1.6 and 3.5 ± 1.5, respectively, demonstrating a statistically significant overall clinical improvement after VPS surgery (*p* < 0.001). Demographic, clinical, and follow-up data are summarized in Table 1.

**Table 1:** Demographic, clinical, and follow-up data of the cohort

	*n* = 76
Mean age (SD)	72.9 (± 9.9)
Sex, female, n (%)	23 (30.3%)
Sex, male, n (%)	53 (69.7%)
Mean follow-up, months, median (IQR)	36 (24–42)
Mean preoperative iNPHGS (SD)	4.2 (± 1.6)
Mean postoperative iNPHGS (SD)	3.5 (± 1.5)

No early postoperative complications were reported, while a total of 7 (9.2%) patients required second surgery due to late complications. Specifically, 2 (2.6%) patients underwent subdural hematoma evacuation, 1 (1.3%) patient required shunt revision surgery for proximal catheter obstruction, 2 (2.6%) patients underwent shunt revision surgery for distal catheter displacement, and 2 (2.6%) patients required externalization of distal catheter due to shunt infection secondary to an abdominal abscess, and a retroauricular wound dehiscence. Overall, 4 (5.2%) patients developed asymptomatic subdural hematoma/hygroma that did not require surgical evacuation. Complete data on early and late postoperative complications are summarized in Table 2.

**Table 2:** Early and postoperative complications of the cohort

Early postoperative complication, n (%)	0 (0%)
Late postoperative complication, n (%)	
Subdural hematoma/hygroma not requiring surgery Subdural hematoma/hygroma requiring surgery Proximal catheter obstruction Distal catheter displacement Shunt infection Valve dysfunction	4 (5.2%) 2 (2.6%) 1 (1.3%) 2 (2.6%) 2 (2.6%) 0 (0%)
Patients requiring second surgery, n (%)	7 (9.2%)

The mean initial valve opening pressure of the DPU unit was 11.3 ± 2.6 cmH_2_O, with a median value of 12 cmH_2_O. The mean initial valve opening pressure of the GU was 20.5 ± 1.5 cmH_2_O, with a median value of 20 cmH_2_O. At the latest follow-up, the mean final opening pressure of the DPU unit was 8.9 ± 3.5 cmH_2_O, with a median value of 8.5 cmH_2_O. Lastly, at the latest follow-up, the mean final opening pressure of the GU was 20.3 ± 1.5 cmH_2_O, with a median value of 20 cmH_2_O. Overall, the differences between the initial and the latest valve settings were – 2.4 cmH_2_O for the DPU unit and – 0.2 cmH_2_O for the GU.

**Fig. 1:**
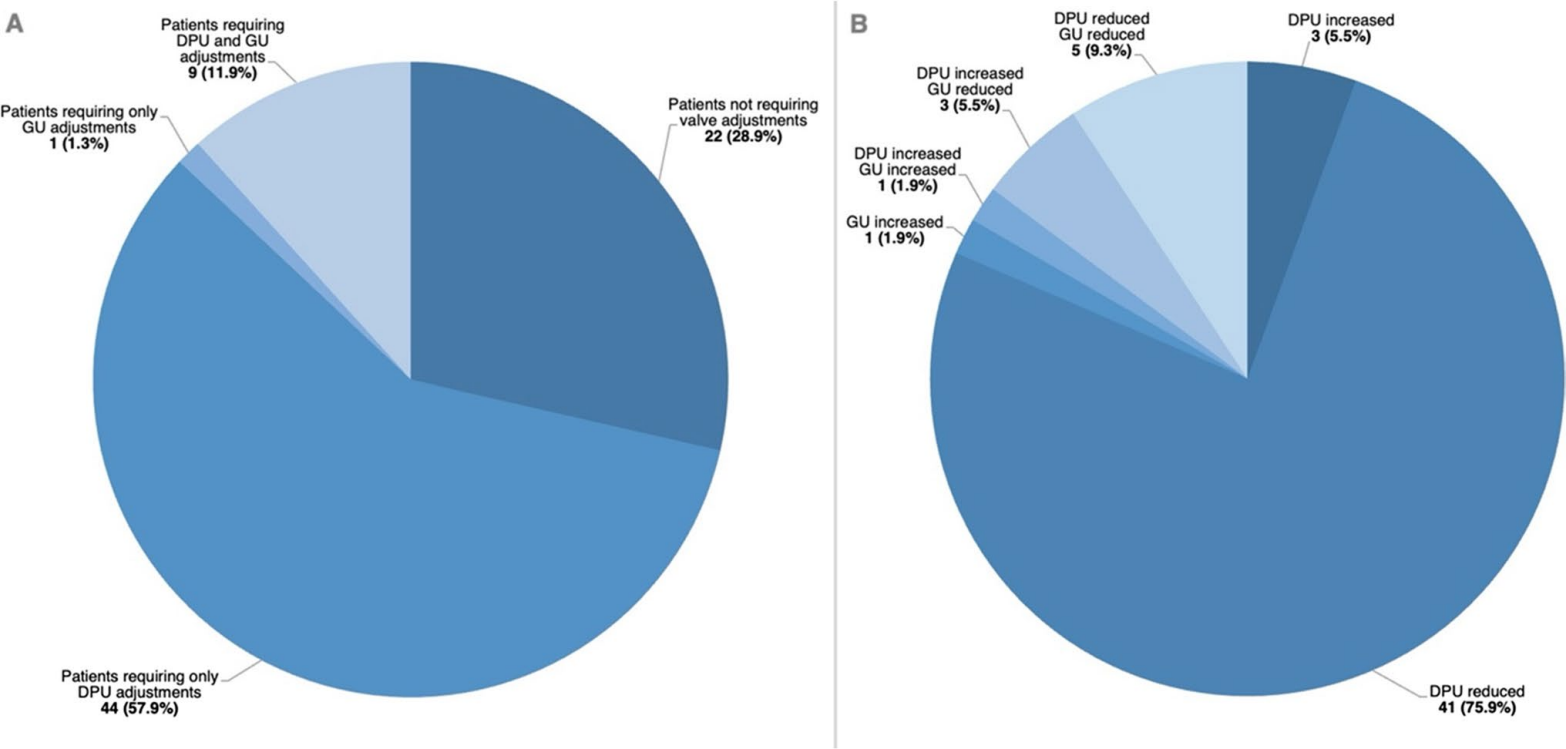
Pie charts reporting (**A**) complete data on number and type of postoperative valve adjustments in the overall cohort, and (**B**) detailed data on frequencies and types of post-implantation adjustments in the group of patients who underwent postoperative setting modifications

A total of 22 (28.9%) patients did not require any valve setting adjustments during postoperative follow-up. Overall, 54 (71.1%) patients underwent postoperatively noninvasive valve setting adjustments due to either suboptimal neurological symptoms management or postoperative complication not requiring surgical intervention, such as asymptomatic subdural hematoma or hygroma. The mean number of post-implantation valve setting modifications was 1.6 ± 1.4, with a median value of 1. Overall, 44 (57.9%) patients required post-implantation adjustments only for the DPU unit, 1 (1.3%) patient required adjustments only for the GU, and 9 (11.9%) patients required adjustments for both the DPU and GU (Fig. 1). More specifically, among the patients requiring only DPU adjustments, 3 (6.8%) underwent an increase in valve opening pressure was obtained, while 41 (93.2%) required a reduction in valve opening pressure to optimize clinical outcome. The single patient requiring exclusively GU adjustments underwent an increase in opening pressure. Lastly, among the 9 patients requiring adjustments to both the DPU and GU, in 1 (11.1%) case both opening pressures were increased, in 3 (33.3%) cases the DPU pressure was increased while the GU pressure was reduced, and in 5 (55.6%) cases both opening pressures were reduced. Complete data on initial and final opening valve pressures, as well as the number and type of post-implantation valve setting adjustments are summarized in Table 3.

**Table 3:** Complete data on initial and final opening valve pressures, number and type of post-implantation valve setting adjustments

Initial valve opening pressure, cmH_2_O	
Mean DPU opening value (median) Mean GU opening value (median)	11.3 ± 2.6 (12) 20.5 ± 1.5 (20)
Final valve opening pressure, cmH_2_O	
Mean DPU opening value (median) Mean GU opening value (median)	8.9 ± 3.5 (8.5) 20.3 ± 1.5 (20)
Mean number of post-implantation valve setting modification (SD) Median number of post-implantation valve setting modification	1.6 (± 1.4) 1
Patients not requiring post-implantation valve setting modification, n (%) Patients requiring post-implantation valve setting modification, n (%) Patients requiring only DPU modification Patients requiring only GU modification Patients requiring both DPU and GU modification	22 (28.9%) 54 (71.1%) 44 (57.9%) 1 (1.3%) 9 (11.9%)
Patients requiring only DPU modification, n (%) Elevation of valve pressure Reduction of valve pressure	*n* = 44 3 (6.8%) 41 (93.2%)
Patients requiring only GU modification, n (%) Elevation of valve pressure Reduction of valve pressure	*n* = 1 1 (100%) 0 (0%)
Patients requiring both DPU and GU modification, n (%) Elevation of DPU pressure + Elevation of GU pressure Elevation of DPU pressure + Reduction of GU pressure Reduction of DPU pressure + Elevation of GU pressure Reduction of DPU pressure + Reduction of GU pressure	*n* = 9 1 (11.1%) 3 (33.3%) 0 (0%) 5 (55.6%)

### Postoperative outcomes

No significant association were found between postoperative iNPHGS scores and age, initial DPU opening pressure, and initial GU opening pressure (*p* > 0.05). Similarly, no significant association were found between the frequency and the number of postoperative opening pressures modifications, and age, initial DPU opening pressure, and initial GU opening pressure (*p* > 0.05). A significant positive linear correlation was found between initial and final DPU opening pressures (R: 0.542, *p* < 0.001). Specifically, a univariate linear regression model demonstrated a significant 0.7 cmH_2_O increase of the final DPU opening pressure for each 1 cmH_2_O increase of the initial DPU opening pressure, suggesting that DPU adjustments were homogenously performed during the follow-up. Lastly, no significant association were found between postoperative overdrainage complications, postoperative complications requiring surgical intervention, and age, initial DPU opening pressure, and initial GU opening pressure (*p* > 0.05). Complete data on postoperative outcomes are reported in Tables 4 and 5.

**Table 4:** Spearman’s Rho correlation tests evaluating the association between postoperative iNPHGS scores, number of valve setting adjustments, final DPU pressure, and age, initial DPU pressure, and initial GU pressure

Variable		Age	Initial DPU pressure	Initial GU pressure
iNPHGS	Rho	0.078	0.181	−0.211
	pValue	0.516	0.134	0.079
Number of adjustments	Rho	−0.190	0.039	0.066
	pValue	0.173	0.788	0.648
Final DPU pressure	Rho	−0.122	**0.542**	0.050
	pValue	0.309	** < 0.001**	0.681

**Table 5:** Mann–Whitney U tests assessing the association between the frequency of valve setting adjustments, postoperative complications requiring surgical intervention, overdrainage complications, and age, initial DPU pressure, and initial GU pressure

	Post-implantation adjustment “No”	Post-implantation adjustment “Yes”	Mean differences	pValue
Age	70.4	74.0	3.6	0.462
Initial DPU pressure	12.0	11.1	0.89	0.283
Initial GU pressure	20.7	20.5	0.22	0.593
	Surgical complications “No”	Surgical complications “Yes”	Mean differences	pValue
Age	72.8	73.4	0.582	0.872
Initial DPU pressure	11.3	11.8	0.561	0.682
Initial GU pressure	20.6	20.0	0.606	0.379
	Overdrainage complications “No”	Overdrainage complications “Yes”	Mean differences	pValue
Age	72.6	75.7	3.09	0.397
Initial DPU pressure	11.4	10.8	0.548	0.672
Initial GU pressure	20.6	20.0	0.600	0.427

## Discussion

The results of this study demonstrated the efficacy and safety of adjustable differential pressure valves with gravitational unit for the surgical treatment of iNPH. After a mean 3-year follow-up, a statistically significant improvement of clinical outcome in terms of iNPHGS scores was demonstrated in the cohort of patients with iNPH treated with VPS using gravitational valves. The overall postoperative complication rate was acceptable and comparable to previous studies. Overdrainage complications were significantly lower compared to analogous studies, with a number of post-implantation valve pressure adjustments in accordance with the evidence from the literature. Thus, the results suggest considering VPS surgery with GV valves a valuable, effective, and safe strategy for the surgical treatment of iNPH.

Historically, the development of CSF shunting valves with a gravitational technology was driven by the necessity to limit postoperative overdrainage complications associated to traditional shunt systems with exclusively differential pressure technology, particularly during the transitions from clino- to orthostatic position. Since the first clinical application of GV in 2005, robust evidence from the literature progressively demonstrated its efficacy and safety in terms of postoperative clinical outcome and complication rate [6]. The “Shunt Valves plus shunt Assistant versus Shunt valves alone for controlling Overdrainage in idiopathic Normal pressure Hydrocephalus in Adults” (SVASONA) was a multicenter, open label, randomized trial conducted in 2013 by Lemcke et al. which demonstrated a significant reduction in the incidence of overdrainage complication in the cohort of patients treated with GV, with superior therapeutic index compared to non-gravitational programmable valves [13]. Thereafter, several studies confirmed the advantages of GV in terms of postoperative neurological outcome and overdrainage complication, such as chronic subdural hematoma or hygroma [7, 10, 21, 25]. Nevertheless, while acknowledging the evidence supporting the use of GV for the treatment of iNPH, to date some issues are yet to be clarified.

The key advantage of GV relies in the ability to finely adjust CSF drainage based on posture aiming to reduce overdrainage-related complications, while maintaining adequate symptom relief. Up-to-date iNPH series reported nontraumatic subdural hematoma rates ranging from 0 to 16% regardless of the type of implanted valve, with cases requiring surgical evacuation described in up to 7% of the cases [6, 11]. In this context, in a recent metanalysis by Giordan et al. on a total of 2461 patients the number of patients with subdural hematoma requiring surgery were almost half with FV compared to FDP valves [6, 15]. Moreover, the addition of GU components to the shunt system led to a decrease by 90% in the rate of subdural hematoma and overdrainage symptoms [23, 26].

Despite its advantages, GV have some drawbacks. Adjustable GV are significantly more expensive compared to FDP valves, thus potentially limiting the healthcare accessibility [1]. In addition, in a recent retrospective study Zipfel et al. emphasized a higher rate of valve dysfunction in the GV group compared to differential pressure valves without antigravitational/antisiphon technology [28]. Despite evidence on GV dysfunction rate is still limited, the potential for higher shunt malfunctioning rates requiring shunt revision surgery along with the significantly higher costs of the valves suggest to carefully evaluate the cost-effectiveness of GV in the treatment of iNPH.

The evidence from our results demonstrates the overall efficacy of GV in treating iNPH, with a statistically significant reduction in postoperative iNPHGS scores after a mean follow-up of 3 years. The rate of postoperative subdural hematoma requiring surgical evacuation was 2.6%, comparable to findings from other recently published case series and metanalyses [2, 6]. Overall, only one (1.3%) patient underwent revision surgery for proximal catheter obstruction. Notably, no cases of valve dysfunction were reported, which significantly differs from other valve dysfunction rates described in the literature reporting revision surgery rates for valve malfunction up to 19% [28].

During follow-up, 54 (71.1%) patients required post-implantation adjustments of valve pressure settings. Despite limited data in the literature on the prevalence of post-implantation valve pressure modifications, our results significantly differ from other studies reporting an adjustment rate of 44.1% [21]. The mean number of post-implantation pressure adjustments in our case series was 1.6, which is comparable to similar studies in the literature reporting 1.7 to 3.1 adjustments to achieve the desired clinical target during the follow-up [16, 20]. More specifically, among the patients who required post-implantation adjustments, 81.4% required adjustments exclusively of the DPU, while modifications of the GU or both the valve components pressures were performed in 1.9% and 16.7% of the cases, respectively. Furthermore, among patients requiring only DPU adjustments, an increase in opening pressure was necessary in only 6.8% of cases, whereas in the remaining 93.2%, a reduction in opening pressure was required to optimize clinical outcomes. In the single patient requiring adjustments exclusively of the GU, an increase in opening pressure was deemed necessary.

Some considerations can be inferred from these results. In most cases, a reduction in the initial DPU opening pressure was necessary to achieve the desired clinical target. Given this, it is plausible to suggest that the availability of valves with an adjustable differential pressure component plays a significant role in optimizing clinical target, rather than lowering the overdrainage complication rate which would require an elevation of the DPU pressure. Furthermore, considering the low overdrainage complication rate in our cohort and the necessity to lower the DPU values in most patients, the presence of a GU in the shunt system appears to be the most significant factor in minimizing postoperative overdrainage complications.

Given the favorable postoperative clinical outcomes and complication rates obtained with relatively high initial DPU and GU opening pressures compared to other studies, it is reasonable to infer that GV may provide a higher therapeutic index than FDP valves for the treatment of iNPH [12, 24, 25]. Lastly, previous studies have reported relatively high rates of valve dysfunction and raised concerns regarding the cost-effectiveness of GVs, limiting their widespread adoption. In contrast, our series demonstrated no cases of valve malfunction, highlighting the safety and reliability of this technology in clinical practice. These findings represent a significant departure from earlier evidence and suggest that GVs may offer a more effective and durable option for the management of iNPH. This novel evidence warrants further investigation to confirm these results and refine the therapeutic potential of GVs in this setting.

### Limitations

This study is a single-center retrospective observational analysis without a prospective control group, which inherently introduces potential selection biases and limits the generalizability of the findings. In this case series, the choice to implant gravitational valves was driven by clinical considerations rather than standardized selection criteria, aiming to evaluate their performance in a clinical setting. Addressing these limitations would require complementary prospective studies with predefined selection criteria and comparative analyses involving different valve types.

## Conclusion

The results of this study suggest that adjustable GVs are a safe and effective alternative to traditional fixed differential pressure valves for the surgical treatment of iNPH. After a 3-year follow-up, the overall postoperative complication rate was acceptable and comparable to other case series and meta-analysis. Our results disproved previously reported high rates of GV dysfunction and confirmed a low rate of postoperative overdrainage-related complications. The high prevalence of postoperative DPU opening pressure modification suggests that the adjustable differential pressure unit seems to be fundamental to tailorize the CSF shunt system dynamics to achieve the optimal clinical outcome. In addition, adding a GU seems to significantly contribute to lower the overdrainage-related complication rate.

Overall, the favorable postoperative complication profile and the low rate of valve dysfunction requiring revision surgery could justify the initial higher costs of the GVs for the healthcare system. Future comparative studies with cost-effectiveness evaluation are mandatory to analyze the applicability of adjustable GVs in the treatment of iNPH.

## Data Availability

No datasets were generated or analysed during the current study.

## Abbreviations

CSF: Cerebrospinal fluid
DPU: Differential pressure unit
FDP: Fixed pressure valve
FV: Flow-regulated valve
GV: Gravitational valve
GU: Gravitational unit
iNPH: Idiopathic normal pressure hydrocephalus
INPHGS: Idiopathic normal pressure hydrocephalus grading scale
TT: Tap test
TUGT: Timed-up and go test
VPS: Ventriculoperitoneal shunt
10MWT: 10-Meter walking test
